# The effect of swine insurance participation on swine production efficiency: Evidence from China

**DOI:** 10.1371/journal.pone.0317759

**Published:** 2025-03-12

**Authors:** Jingyue Feng, Shan He, Chunli Wan, Jia Liu, Fengjie Xie

**Affiliations:** 1 College of Economics and Management, Shenyang Agricultural University, Shenyang, Liaoning, China; 2 Liaoning Province Agricultural Development Service Center, Shenyang, Liaoning, China; Indian Institute of Management Bodh Gaya, INDIA

## Abstract

How does swine insurance affect the swine production efficiency in China? We focus on micro-survey data from 582 swine farmers in Liaoning Province, and uses the propensity score matching method (PSM) and mediated effects model for the empirical examination. The results indicate that swine insurance positively impacts production efficiency, compared to uninsured farmers, those who participate in swine insurance exhibit a 4.7% improvement in production efficiency. Additionally, the estimations from the mediated effects models indicate that swine insurance significantly enhances swine production efficiency by influencing risk appetite, production decision and technology adoption. Furthermore, the heterogeneity analysis revealed that the positive effect of swine insurance on production efficiency becomes more pronounced as the scale of farmers’ swine production expands. Apart from this, the correlation between swine insurance coverage level and production efficiency reveals a significant U-shaped curve. These findings provide valuable insights for improving the swine insurance system and fostering the growth of the swine industry.

## 1. Introduction

Uncertainty in production and price is a prevalent issue in agriculture on a global scale [1], and swine production is not immune to this challenge. Swine farming serves as a key source of income and is essential for maintaining food and nutritional security for a significant part of the world’s population [2]. However, the production of swine is constrained by various factors including the migration of rural labor, continuous increases in feed costs, extreme weather changes, and stringent environmental regulations [3,4]. Furthermore, the worldwide swine industry is now dealing with the unparalleled threat of African swine fever (ASF), which initially identified in Africa, spread rapidly and extensively to Armenia, Azerbaijan, Russia, and 23 European countries by the end of June 2023, having posed a significant threat to the whole world [5]. Under these circumstances, swine insurance becomes particularly essential for mitigating production risks and enhancing farmers’ resilience. It provides essential functions such as pre-disaster prevention and post-disaster compensation, helping to stabilize farmers’ income [6], raise welfare in the whole rural communities, effectively minimize the effect of natural disasters and disease risks on agricultural output [7]. These mechanisms ensure farmers are better prepared to manage unforeseen events and maintain financial stability.

Enhancing production efficiency is essential for boosting the profitability of the swine industry, as it ensures a stable domestic food supply and contributes to social stability [2]. By lowering production costs and improving quality, greater efficiency directly stimulates rural economic development and increases farmers’ incomes. Moreover, stable production efficiency helps to mitigate food safety risks associated with production variability. The experiences of developed countries provide valuable insights. For instance, since the 1970s, the United States has achieved high swine production efficiency through specialization, economies of scale, and contract farming [8]. Similarly, Spain has improved production efficiency by enhancing average daily weight gain, feed conversion rates, and breeding technologies. However, despite significant progress in production efficiency, these countries still face challenges such as labor shortages, rising costs, and environmental issues [9].

China is currently the world’s largest producer and consumer of pork, and also holds the distinction of having the largest agricultural insurance market by scale. Swine production plays a significant role in China’s economy, with swine farming developing into a major industry generating over one trillion CNY annually. In 2023, swine production reached 57.94 million tons, which represented approximately 54.89% of the global production.. However, the emergence of ASF and the COVID-19 pandemic caused significant fluctuations in China’s swine prices and production between 2019 and 2021. Although China leads in global swine production, it currently bears the highest average production costs among the major pork-producing countries. Additionally, China’s swine production efficiency remains significantly lower than that of other leading pork producers [10]. Consequently, improving China swine production efficiency is crucial. In addition to being a leading swine producer, China holds the position as the leading agricultural insurance market worldwide. In 2021, the agricultural insurance premiums in China totaled CNY 96.518 billion, maintaining its position as the highest in the world for two consecutive years [11]. This strong insurance market has played a crucial role in stabilizing the agricultural sector, providing farmers with a safety net to manage such disruptions.

Previous research indicates that agriculture insurance enhances production efficiency by diversifying risk [12] and securing income [13]. It plays a vital role in maintaining the availability of agricultural products and providing stability to farmers’ earnings, and encourages the adoption of new technologies [14], however, the majority of research has focused on crop farmers, with only a few studies examining the livestock sector [15]. Livestock insurance is significantly more intricate and encounters more obstacles compared to crop insurance [6]. Despite this, it has garnered less attention and research compared to crop insurance.

This paper utilizes micro-survey data from 582 swine farmers in Liaoning Province in China in 2023, with propensity score matching (PSM) method and mediated effects model to empirically examine the impact of swine insurance participation on production efficiency, as well as the underlying transmission mechanisms, and whether this effect varies according to different scale of farms. This research makes several contributions. Firstly, it expands the research on the microeconomic effects of swine insurance from the perspective of resource allocation, further explores the underlying mechanisms and examines the impact of swine insurance on production efficiency under varying scales of operation. Existing studies on swine insurance predominantly focus on its impact on income and the factors influencing farmers’ participation [16], while neglecting the changes in resource allocation following insurance enrollment. By examining how swine insurance affects farmers’ risk appetite, production decision, and technology adoption to ultimately enhance production efficiency, this study introduces a new perspective and empirical evidence to the literature on livestock insurance. Secondly, it further extends the research boundaries on swine production efficiency, provides a foundation for developing a more comprehensive theoretical framework. While previous studies have predominantly focused on the relationship between inputs and outputs, this research broadens the theoretical and empirical scope by incorporating risk management practices such as swine insurance. It highlights the critical role of financial instruments and insurance policies in the swine production process and their impact on enhancing production efficiency. Lastly, it uncovers the nonlinear relationship between swine insurance and production efficiency, offers valuable guidance for optimizing the design of insurance coverage levels in the future. By exploring the nonlinear dynamics between swine insurance coverage levels and production efficiency, this research reveals how differing coverage levels impact the risk protection capacity offered to farmers, leading to varying effects on their production efficiency. This investigation sheds light on previously underexplored dynamic complexities in the field.

## 2. Literature review

### 2.1. Research on agricultural insurance

Agricultural insurance is vital to agricultural production worldwide [17]. Back to 1992, Hazell found that agricultural insurance is helpful in the developing countries, especially in assisting low-income rural households in coping with income losses caused by catastrophe [18]. Several studies have examined how crop insurance influences the adoption of new technologies and agrochemical input [19]. There is substantial evidence indicating that crop insurance positively influences farmers’ willingness to adopt new technologies [19–21], and alters farmers’ behavior, encouraging them to produce crops that carry higher risks but offer higher returns [20,22]. In addition, agricultural insurance has been demonstrated to potentially enhance the financial stability of both lenders and farming operations [23], the combination of insurance and the credit market ease risk constraints and result in increased investment [23,24]. Numerous studies have also concentrated on how the crop insurance program affects farmers’ acreage decisions [25–27], a study conducted in Mali reveals that the introduction of area-yield insurance for cotton led cotton farmers to expand their cultivated area and increase input purchases by roughly 25–40 percent [22].

Although there is extensive research on agriculture insurance globally, most studies focus on crop farmers, with only a few addressing livestock farming, particularly swine production. Dairy cattle insurance has evolved significantly over the years, Valvekar (2011) [28] investigated cattle farmers’ risk preferences and factors that influence their incentives to adopt cattle insurance, Zhang and Zhao (2017) [29] found the dairy cattle insurance participation significantly changed farmers’ behaviors, and incentivized them to expand the scale of farming. In regions like Australia and New Zealand, where sheep farming is a significant part of the agricultural economy, livestock insurance has been widely adopted to mitigate these risks. The introduction of index-based livestock insurance (IBLI) has been a notable advancement, providing coverage based on predefined indices such as rainfall or pasture availability rather than actual losses [30]. Aquaculture insurance is less common but has gained attention due to the high value and vulnerability of fisheries. Roll (2019) [31] found that the Norwegian salmon insurance has an enhancing effect on both production and efficiency.

For swine insurance, Cai et al. (2009) discovered that offering formal insurance access significantly boosts farmers’ inclination to raise sows [32]. Rao and Zhang (2020) used data from Deqing county in China to discover that participation in insurance increases the likelihood of farmers reporting animal diseases to the government, which assists the government in the prevention and surveillance of animal disease outbreaks [33]. The United States, as the world’s second-largest swine producer, was the first country to implement swine insurance, with its origins tracing back to the 1930s. The U.S. has developed a highly mature and comprehensive swine insurance system, including livestock risk protection (LRP), which provided by insurance companies, offers price risk protection and essentially functions as a put option, and livestock gross margin (LGM), which insures against losses in production margins, acting as a bundled option. Research indicates that with a 50% government subsidy, the proportion of insured LGM farmers increases significantly [34,35], subsidies effectively boost participation rates in LRP insurance, reduce producer costs, stabilize income, and mitigate risks [36]. In several developed western European countries, such as Germany and France, government-subsidized commercial insurance models are employed. These programs are primarily operated by cooperative insurers, with premiums partially subsidized by the government [37]. In Italy, livestock insurance has been shown to reduce the likelihood of income decline, with farms managed by younger farmers appearing to face greater risks [38]. The insufficient research on swine insurance necessitates further attention and in-depth studies to provide more comprehensive risk management strategies and policy support for the swine industry.

### 2.2. Research on swine production efficiency

Swine production efficiency, a crucial indicator for evaluating industrial performance and competitiveness, acts as a fundamental benchmark for gauging the high-quality development achieved within the swine industry. Labajova et al. (2016) used multidirectional efficiency analysis to calculated technical efficiency in Sweden, and explain how different farm-specific characteristics affect the efficiency [39]. Hu and Yu (2022) use the Tobit model, which ensures more accurate estimations of relationship between limited dependent variable and independent variables, to examine how epidemic prevention and control positively influence production efficiency, and discovered that disease management significantly enhances efficiency, particularly in large-scale farming operations [40]. Some researches explore the factors influencing swine production efficiency. Ji et al. (2023) used the slack-based measure (SBM) model to estimate the swine production efficiency of China, and determined that the key to boosting the efficiency lies in encouraging the adoption of advanced technologies, including those that save labor, reduce material usage, and cut emissions [41]. Wang (2011) measured swine production efficiency across 15 major swine-producing provinces in China, indicate that feed input is the most significant factor affecting swine output [42]. Similarly, Wang (2021) also identified material inputs, especially feed, as the primary drivers of improved swine production efficiency, underscoring the critical importance of material inputs [43].

### 2.3. Research on the impact of agricultural insurance on production efficiency

At present, there is a scarcity of research on the impact of agricultural insurance on production efficiency, as most studies concentrate on crop insurance. Existing research suggests that agricultural insurance can effectively encourage farmers to optimize factor allocation in agricultural production through mechanisms like cost reduction, reducing moral hazard, and adjusting resource distribution [44], thereby enhancing production efficiency. Fu et al. (2022) found that agricultural insurance improves production efficiency by promoting land transfer to expand scale, increasing capital investment through the synergistic effect of agricultural insurance and credit, and improving technical investment through strengthened agricultural technology training [45]. Similarly, Ren (2021) found that higher levels of agricultural insurance coverage can stimulate short-term investment growth, enhance the specialization of crop structures, and improve loan accessibility, thereby increasing agricultural production efficiency [46]. Moreover, certain studies have investigated how agricultural insurance affects production efficiency considering environmental effects. Li et al. (2024) showed that green finance can significantly enhance agricultural green total factor productivity, displaying an inverted U-shaped relationship and clear regional disparities [47]. Fang et al. (2021) found that crop insurance significantly increases agricultural green total factor productivity, which amplified by the adoption of green agricultural technologies, including precision sowing, deep fertilization, subsoiling, and no-tillage practices [48].

In summary, existing research on the effects of agricultural insurance on production efficiency mainly focuses on the planting sector. In contrast, the swine industry, a crucial component of agriculture and the rural economy, has received comparatively less attention. Previous research on swine production efficiency has primarily examined the input-output relationship, emphasizing how inputs such as feed, labor, and technology influence meat yield and quality, it neglects the influence of financial instruments, particularly insurance. In addition, current studies on swine insurance mainly concentrate on its effects on income and the factors influencing farmers’ insurance participation [16], lacking an exploration of changes in resource allocation after farmers adopt insurance. Clarifying the role of swine insurance on swine production efficiency is of significant theoretical and practical importance. It helps to foster the high-quality development of the entire swine industry and assess the effectiveness of existing swine insurance policies.

## 3. Policy background and theoretical analysis

### 3.1. Policy background on China swine insurance

China stands as the largest producer and consumer of pork globally, making swine production a critical component of its agricultural sector and overall economy. In 2023, the total number of pigs slaughtered in China reached 726.62 million, resulting in a pork production output of 57.94 million tons. Swine industry supports the livelihoods of millions of farmers in China. Ensuring stability and growth in this sector can significantly influence rural incomes. The swine industry in China is highly vulnerable to natural disasters and diseases such as ASF, which can cause massive economic losses, effective insurance mechanisms can help stabilize incomes and encourage investment in better farming practices. Compared to countries like the United States, which have a longer history of swine insurance development, research on swine insurance in China began relatively late. Notably, significant studies in this area only started to emerge after 2007, this timing aligns with the formal pilot implementation of policy-based swine insurance in the country.

**Fig 1:**
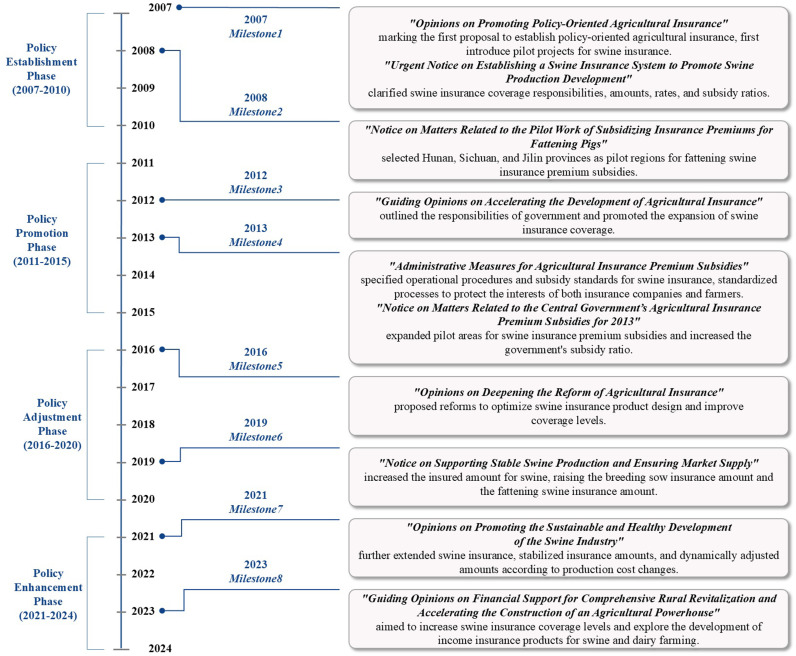
Review of the main government documents on swine insurance policies in China.

Based on a review of the main government documents on swine insurance policies since 2007 (Fig 1), evidence suggests that the Chinese government is consistently refining the swine insurance system and policies. These improvements bolster farmers’ capacity to handle market and natural risks, promoting the stability and sustainable development of swine production.

### 3.2. Theoretical analyses and research hypotheses

#### 3.2.1. Swine insurance and production efficiency.

Swine insurance works directly on production efficiency through both ex ante and ex post effect. On one hand, there is risk prevention in the ex ante effect. The swine insurance company regularly promotes and disseminates scientific breeding knowledge and disease prevention to farmers. These preventive measures can help farmers reduce potential risks, thereby decreasing the likelihood of significant losses and helping them enhance production levels and optimize production efficiency. On the other hand, there is disaster compensation after a disaster, which known as ex post effect [49]. In the event of a disaster, swine insurance shifts the risk from farmers to the insurance company, greatly reducing the economic losses suffered by farmers and stabilizing their expected income. This enables farmers to maintain continuity and stability in production, avoiding reductions in investment or production scale. Additionally, through insurance compensation, farmers receive direct economic reimbursement, quickly helping them resume production. This avoids production interruptions due to insufficient funds, thereby improving production efficiency. This analysis proposes Hypothesis 1.

H1: Farmers’ participation in swine insurance can increase swine production efficiency.

#### 3.2.2. Mediating effects of risk appetite.

Purchasing swine insurance disperses and transfers risks, effectively alters farmers’ risk appetite [50], thereby increasing their willingness to adopt new opportunities and engage in proactive production, ultimately improving overall swine production efficiency. Risk appetite refers to the subjective psychological attitude of farmers when facing risks in swine production and management [51], swine insurance’s compensation mechanisms greatly reduces the impact of natural disasters and disease outbreaks on farmers’ production and stabilizes expected returns [52]. Adopting swine insurance increasing farmers’ risk tolerance and acceptance, changes their risk appetite [53]. Risk appetite is a key factor in their decision-making contexts [51]. According to prospect theory, constrained by uncontrollable production risks and information asymmetry, farmers’ risk appetites vary significantly, leading to differences in decision-making [54]. For example, when facing uncertainty in expected returns, farmers with strong risk appetites generally exhibit a more proactive attitude and are more inclined to embrace new opportunities [55], such as expand their production scale, thereby further improving production efficiency. In contrast, farmers with weaker risk preferences often adopt a cautious attitude [56], making it difficult to improve product quality and prices, hindering production efficiency. This analysis proposes Hypothesis 2.

H2: Farmers’ participation in swine insurance can increase swine production efficiency by enhancing risk appetite.

#### 3.2.3. Mediating effects of production investment.

Swine insurance programs can influence farmers’ investment decisions by reducing risk and easing capital constraints [27,57]. On one hand, Farmers frequently face uncertainty in production and pricing, leading to unexpected fluctuations in output quantity or market price due to various factors, including natural disaster or epidemic disease, at the same time, the costs of feed rise sharply, so that deter farmers to invest in expanding scale and upgrading techniques. Studies ﬁnd that the provision of insurance improves farmers’ resilience to risks and changes behavior toward profitable but risky investments, and increases incentives for farmers to enhance financial inputs into production [22,58,59]. On the other hand, swine insurance helps farmers obtain loans and increase access to finance. The insured swine can act as direct or implied collateral, thereby increasing the likelihood of loan approval [6]. Farmers face credit limit and financing constraints because of lacking collateral, and are generally marginalized by formal financial institutions in rural areas when applying for loans. The integration of insurance and credit facilities greatly enhances farmers’ access to financial support for investment. This reduces the need for precautionary savings, allowing farmers to allocate more cash towards production activities [60]. The mechanism talked above effectively alleviates capital constraints, as investment increases, farmers may expand their production scale, achieving economies of scale, which in turn reduces unit costs and enhances overall production efficiency [50,61]. This analysis proposes Hypothesis 3.

H3: Farmers’ participation in swine insurance can increase swine production efficiency by enhancing production investment.

#### 3.2.4. Mediating effects of technology adoption.

Swine insurance can effectively distribute farmers’ risk and have a behavior-altering stimulation effect on technology adoption decisions [62]. As farmers are expected utility maximisers [63], they will weigh the risks of adopting new technologies against their personal risk tolerance and ability to withstand adversity when making technology adoption decisions. The frequent and severe fluctuations in swine prices have increasingly become a significant risk factor, the application of technology will further increase the uncertainty of returns. Uninsured farmers might opt to continue using low-risk, low-productivity technologies due to the absence of both ex ante and ex post risk management strategies [20,64]. Swine insurance provides a mechanism for risk transfer, enhancing farmers’ risk tolerance, which allows them to allocate more resources to high-risk investments, such as adopting new technologies, thereby promoting specialization and modernization. According to the new economic growth theory [65], the contribution of technology adoption to production efficiency is significant, whose findings are consistent with the swine production field. Technology adoption optimizes production processes, reduces reliance on manual labor, and lowers labor costs, thereby improving production efficiency and driving the advancement of the entire swine industry [49]. This analysis proposes Hypothesis 4.

H4: Farmers’ participation in swine insurance can increase swine production efficiency by stimulating technology adoption.

**Fig 2:**
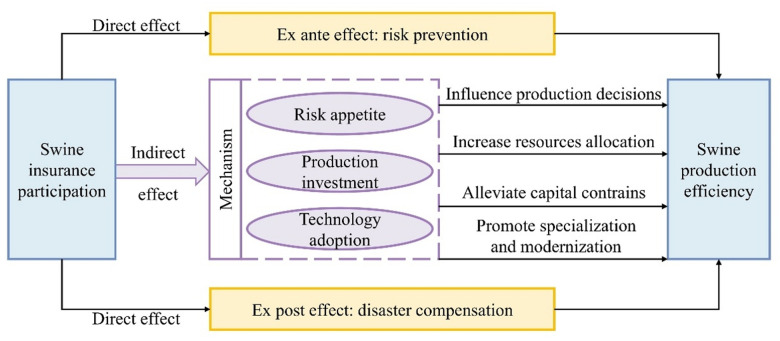
Conceptual framework of the effect of swine insurance participation on swine production efficiency.

The analyses constructing the conceptual framework of this research is displayed in Fig 2.

## 4. Materials and methods

### 4.1. Data source

The data for this paper were collected from a micro-survey of 582 swine farmers conducted from May to November 2023 in Kangping County (Shenyang), Haicheng and Taian counties (Anshan), Tieling and Changtu counties (Tieling), and Yixian County (Jinzhou) in Liaoning Province. Liaoning Province is located in the southern part of Northeast China, which coordinates Between 118°53’E to 125°46’E longitude and 38°43’N to 43°26’N latitude. Liaoning Province plays a significant role in China’s swine industry as a major production and supply region, designated as a key area with potential for growth. Several leading industrial enterprises have established production and processing bases in the region. The development of swine production here not only meets local demand but also supplies for major cities such as Beijing and Tianjin. Additionally, Liaoning Province is a major crop-producing area in China, providing abundant feed resources. The province boasts a well-developed swine industry chain, including feed production, breeding, slaughtering, processing, and marketing. This comprehensive industry chain provides a solid foundation for the development of swine farming in the region.

To ensure the representativeness and validity of the sample, this study adopted stratified sampling combined with randomized selection. Five towns were randomly selected from each county, and 20-25 households were randomly chosen from each town, a total of 637 questionnaires were collected, and after excluding invalid responses, 582 valid questionnaires were retained. The surveys were conducted primarily through one-on-one interviews, for farmers unable to attend due to time constraints, telephone interviews were conducted to ensure data completeness and coverage. The questionnaire included questions across five dimensions: insurance participation status, individual characteristics, household characteristics, farming characteristics, and social factors. To maintain scientific rigor and consistency during the data collection process, strict quality control measures were implemented. Prior to the formal survey, all team members underwent extensive training sessions on the questionnaire to ensure uniform standards in the interviewing process.

### 4.2. Variable selection

#### 4.2.1. Dependent variable.

Data Envelopment Analysis (DEA) is a nonparametric technique used to assess the relative efficiency of decision-making units (DMUs) by solving mathematical programming problems, rather than relying on a predefined production function. Traditional DEA models define an efficient DMU as one that maximizes output with a given input or minimizes input with a given output. Unlike traditional DEA models, the Slack-Based Measure (SBM) model is non-radial and non-angular, incorporating slack variables into the objective function for a more comprehensive efficiency evaluation.

To address the limitations of traditional DEA models and to resolve the issue of ranking efficient decision-making units, this paper employs the Super Efficiency SBM model. Consider that there are n decision-making units, each possessing *m* inputs, *λ* is the weight for inputs and desirable outputs, X and Y represent the input and the desired output, *θ* represent the swine production efficiency. Thus, the SBM model can be mathematically expressed as follows:


$$\begin{array}{c} min\theta =\frac{\left(1+\frac{1}{m}\sum_{i=1}^{m}\frac{X_{i}^{'}}{X_{ik}}\right)}{\left(1-\frac{1}{s}\sum_{r=1}^{s}\frac{Y_{r}^{'}}{Y_{r0}}\right)}\# \end{array}$$ (1)

$$\begin{array}{c} s.t.\overset{N}{\underset{i=1,\neq n}{\sum }}\left(X_{mi}\lambda_{i}-X_{m}^{\text{'}}\right)⩽0\left(m=1,2,\cdot \cdot \cdot ,M\right)\# \end{array}$$ (2)

$$\begin{array}{c} \overset{N}{\underset{i=1,\neq 0}{\sum }}\left(Y_{si}\lambda_{i}+Y_{s}^{\text{'}}\right)⩾0\left(s=1,2,\cdot \cdot \cdot ,S\right)\# \end{array}$$ (3)

The inputs and desirable outputs are shown in Table 1.

**Table 1:** Swine production efficiency indicator system.

Indicator type	Indicator	Description	Unit
**Input**	Labor force	Number of days of household and hired labor	Days
	Technology Input	Depreciation cost of technological equipment for farmers	RMB
	Breeding stock Input	Cost of purchasing breeding stock	RMB
	Feed Input	Feed input cost	RMB
	Vaccine Input	Vaccine and veterinary medicine input cost	RMB
	Capital Input	Depreciation cost of original farm building	RMB
	Maintenance Input	Equipment repair and maintenance cost	RMB
	Energy Input	Water and electricity expenses	RMB
**Desirable output**	Total revenue	Farmers’ total swine breeding income	RMB

#### 4.2.2. Independent variable.

The swine insurance in this paper aims to reduce the losses due to the death of insured pigs during the insurance period, the risk prevention focus mainly on mitigating the risk of swine mortality. In line with Tang and Luo (2021) [66], swine insurance participation is a binary variable, assigned a value of 1 if farmers participate swine insurance and 0 if they do not. This variable was assessed through a questionnaire item: “Did the farmer purchase agricultural insurance in the past year?” with responses coded as yes=1 and no=0”.

#### 4.2.3. Mediating variables.

The mediating variables include risk appetite, production investment, and technology adoption. Risk appetite represents farmers’ personal risk evaluations and significantly impacts their production choices. For example, farmers with a higher risk tolerance may incorporate more risk factors into their swine farming practices. This paper assesses risk appetite according to respondents’ attitudes toward yield uncertainty, following the approach recommended by Menapace et al. (2016) [67]. The assessment involves asking respondents to choose among the following investment options: “1. Low risk with corresponding low return and minimal loss; 2. Moderate risk with moderate return and loss; 3. High risk offering high return but also high potential loss.” The paper adopts the method according to Nie (2013) to measure production investment indicators for farmers [68], which includes feed cost, vaccine cost and other capital cost, specifically, we take the logarithm of all productive investments. According to Fu et al. (2022) [45], the paper measure the indicator of technology adoption by summing the number of farming techniques adopted. In the questionnaire, we inquired about the adoption of various farming techniques within households, the specific techniques including feed formulation technology, breeding technology, manure management technology, and information-based farming technology and many others technologies.

#### 4.2.4. Control variables.

Building on the results of prior research [44,69], this paper includes control variables from multiple perspectives, such as individual characteristics, (age, gender, health status and education level), household characteristics (farming years, distance from the township, specialization level and previous year’s calamities), social characteristics (contacts numbers, social experience, communications with feed companies and frequency of training). Differences in natural resources, market environments and policy implementation may exist across regions. Therefore, this study incorporates regional variable to enhance the robustness of the analysis [50].

The selection of control variables was guided by existing theoretical and empirical research [44,69]. These variables were chosen for their established relevance to either swine insurance participation or production efficiency. For example: highly educated and healthy farmers tend to be more physically capable of engaging in farming. They often possess stronger analytical skills, enabling them to understand and adopt insurance policies and new technologies more quickly, thereby influencing production efficiency. Farmers with extensive years of farming experience are generally better prepared for risks due to their accumulated knowledge and expertise, which may reduce their willingness to participate in insurance. In addition, farmers who have experienced disasters tend to have a deeper understanding of risks, making them more inclined to purchase insurance to mitigate future uncertainties. The inclusion of these control variables enhances the robustness of the research’s findings. Table 2 provides an overview of the descriptive statistics for all variables based on raw data (without truncation or standardization).

**Table 2:** Variable definition and descriptive statistics.

Variable	Definition	Obs	Mean	Std. Dev.	Min	Max
Swine production efficiency	Calculated using SBM, the indicators are shown in Table 1.	582	0.375	0.182	0.258	3.213
Swine insurance participation	Whether the farmer purchased swine insurance in the past year, yes = 1, no = 0	582	0.653	0.476	0.000	1.000
Risk appetite	If you were to invest, which type of investment would you choose? 1. Low risk with corresponding low return and minimal loss; 2. Moderate risk with moderate return and loss; 3. High risk offering high return but also high potential loss.	582	1.540	0.752	1.000	3.000
Production investment	The logarithm of how much you spent in swine production last year, which including feed cost, vaccine cost and other capital cost	582	12.757	1.204	8.355	16.742
Technology adoption	How many farming techniques you adopted in the swine production	582	2.809	1.393	0.000	7.000
Age	Actual age of main production decision makers (years)	582	51.043	8.887	28.000	76.000
Gender	If the main production decision maker is male = 1, female = 2	582	1.100	0.300	1.000	2.000
Health status	Health status of production decision makers: very unhealthy = 1, relatively unhealthy = 2, fair = 3, relatively healthy = 4, very healthy = 5	582	3.897	0.821	1.000	5.000
Education level	Educational attainment of main production decision makers (years)	582	9.014	1.863	0.000	16.000
Farming years	Years of experience in pig farming(years)	582	10.928	5.641	1.000	30.000
Distance from the township	Distance from the family’s place of residence to the nearest township (meters)	582	97.261	339.139	0.000	4000.000
Specialization level	The proportion of pig farming income to total household income.	582	92.575	14.233	3.537	100.000
Previous year’s calamities	Whether faced a natural disaster in the past year, drought = 1, flood = 2, no disaster = 3	582	1.413	0.684	1.000	3.000
Contacts numbers	Number of contacts in WeChat and other social media	582	216.313	125.509	30.000	1112.000
Social experience	Ordinary farmer = 1, private business owner = 2, migrant worker = 3, military personnel = 4	582	1.196	0.592	1.000	5.000
Communications with feed companies	Whether the farmer communicate feeding skills with the feeding companies r, yes = 1, no = 0	582	0.510	0.500	0.000	1.000
Frequency of training	Time of training by the insurance companies, government and epidemic prevention workers last year	582	2.753	1.187	1.000	5.000
Regional variable	Changtu county = 1, Haicheng county = 2, Kangping county = 3, Taian county = 4, Tieling county = 5, Yixian county = 6	582	3.454	1.715	1.000	6.000

### 4.3. Model

#### 4.3.1. Baseline model.

This paper starts by estimating Equation 4 to evaluate the effect of swine insurance participation on swine production efficiency, using an Ordinary Least Squares (OLS) multiple linear regression model.


$$\begin{array}{c} Y_{i}=\beta_{0}+\beta_{1}Insurance_{i}+\gamma Controls_{i}+\varepsilon_{i}\# \end{array}$$ (4)

Where *Y* denotes the swine production efficiency, $Insurance_{}$shows whether the farmer purchases swine insurance, $\beta_{0}$ and $\beta_{1}$ are the intercept term and the coefficient of ${\text{Insurance}}_{i}$, respectively, $Controls_{i}$ signifies the control variables affecting swine production efficiency, *γ* is the coefficients of the control variables, *i* represents the swine farmer, and *ε* indicates the random error term.

#### 4.3.2. Propensity score matching method.

The Propensity Score Matching (PSM) method has been extensively applied in various fields using cross-sectional data, such as labor economics, population economics and agriculture economics [70–74], and is particularly effective between treatment and control groups in observational data [75]. This research adopts PSM for several reasons: Firstly, farmers’ participation in swine insurance is voluntary and self-determined, which introduces sample self-selection bias. Secondly, due to variations in individual resources, participation in swine insurance may lead to selective bias in production efficiency. Finally, issues such as omitted variables, including unobservable factors like individual skills and abilities, could contribute to endogeneity problems. PSM addresses these concerns by constructing a counterfactual control group.

This paper employs the PSM method to examine how farmers’ participation in swine insurance affects their production efficiency. By creating a counterfactual control group that closely resembles the treatment group, we approximate the random assignment of the treatment variable, rendering the participation behavior in swine insurance nearly random. This approach allows us to match the treatment and control groups to examine the effect of swine insurance participation on production efficiency under identical external conditions. The analysis proceeds as follows: Firstly, we match the treatment and control groups. To guarantee the robustness of the model’s matching results, we apply several matching methods, such as k-nearest neighbor matching, caliper matching, and kernel matching, followed by conducting balance tests. Secondly, we compute the Average Treatment Effect on the Treated (ATT) to determine the difference in production efficiency between the treatment and control groups. This difference highlights the effect of participating in swine insurance on production efficiency by comparing the actual outcomes of insured farmers with their counterfactual outcomes. The expression for ATT is as follows:


$$\begin{array}{c} ATT=E\left(Y_{1i} |R_{i}=1\right)-E\left(Y_{0i} |R_{i}=1\right)=E\left(Y_{1i}-Y_{0i} |R_{i}=1\right)\# \end{array}$$ (5)

In Equation 5, ATT represents the Average Treatment Effect on the Treated for farmers participating in swine insurance. $Y_{1i}$denotes the production efficiency of farmers in the treatment group, while $Y_{0i}$represents the production efficiency of farmers in the control group. The term $E\left(Y_{1i}-Y_{0i} |R_{i}=1\right)$signifies the observable average production efficiency for those participating in swine insurance. Conversely, $E\left(Y_{0i} |R_{i}=1\right)$, representing the counterfactual outcome, cannot be directly observed. This counterfactual outcome is estimated using the Propensity Score Matching method to construct an appropriate substitute indicator.

#### 4.3.3. Mediation effects model.

The mediation effect model provides a systematic framework for analyzing the mechanisms through which mediating variables influence the dependent variable. It is particularly suited for exploring potential causal pathways [76] and has been extensively applied in mechanism analysis within economics [50]. This model allows us to investigate the indirect effects of swine insurance on production efficiency, offering a more comprehensive understanding of the impact pathways.

The stepwise method is frequently employed due to its simplicity and the ease with which results can be interpreted. However, it has a significant limitation: it treats the mediator variable in a similar way to a control variable while also including the main independent variable in the model. This approach may result in the problem of “improper control”, making it difficult to achieve consistent estimation results. According to Jiang (2022) [76], the main emphasis should be on examining how independent variables affect the mediating variables, as outlined in Equation 6.


$$\begin{array}{c} M_{i}=\theta_{0}+\theta_{1}Insurance_{i}+\delta Controls_{i}+\mu_{i}\# \end{array}$$ (6)

Mstands for the mediating variables, which include factors risk appetite, production investment, and technology adoption, $\theta_{0}$ is intercept, $\theta_{1}$and *δ* are the corresponding coefficients; and *μ* is the random disturbance term.

## 5. Results

### 5.1. Baseline regression

Table 3 presents the marginal effects of participating in swine insurance on production efficiency. Column (1) presents the results with only swine insurance participation included, showing a positive and statistically significant coefficient of 0.053 at the 1% level. This suggests that adopting swine insurance enhances production efficiency. To ensure the robustness of these findings, control variables are progressively introduced in columns (2) to (4) of Table 3. The coefficients for swine insurance participation after adding these control variables sequentially are 0.044, 0.031, and 0.026, respectively, all positive and statistically significant at the 1% and 5% levels. This confirms the robustness of the regression results. Notably, the coefficients decrease as more control variables are added. This pattern suggests that the positive effect of swine insurance participation on production efficiency may be overestimated if relevant variables are not properly accounted for. In Column (5), after introducing regional variable, the regression coefficient remains significantly positive at the 1% level.Thus, farmers’ involvement in swine insurance boosts production efficiency, confirming Hypothesis 1

**Table 3:** Results of baseline regression.

	(1)	(2)	(3)	(4)	(5)
Swine insurance participation	0.053***	0.044***	0.031***	0.026***	0.025***
	(0.012)	(0.013)	(0.011)	(0.010)	(0.009)
Age		−0.002	−0.001	−0.001	−0.001
		(0.013)	(0.001)	(0.001)	(0.001)
Gender		0.025	0.028	0.024	0.023
		(0.025)	(0.023)	(0.020)	(0.020)
Health status		0.005	−0.001	−0.004	−0.003
		(0.007)	(0.006)	(0.007)	(0.008)
Education level		0.012	0.004	0.001	−0.001
		(0.008)	(0.008)	(0.008)	(0.008)
Farming years			0.001	0.001	0.001
			(0.002)	(0.002)	(0.002)
Distance from the township			0.001***	0.001***	0.001***
			(0.001)	(0.001)	(0.001)
Specialization level			0.001***	0.001***	0.001***
			(0.001)	(0.001)	(0.001)
Previous year’s calamities			0.055**	0.037	0.036
			(0.022)	(0.022)	(0.023)
Contacts numbers				0.001**	0.001***
				(0.001)	(0.001)
Social experience				0.021 *	0.020
				(0.012)	(0.012)
Communications with feed companies				0.023***	0.022**
				(0.009)	(0.008)
Frequency of training				0.023**	0.023**
				0.010	(0.010)
Constant	0.341***	0.269 *	0.115	0.093	0.107
	(0.005)	(0.151)	(0.129)	(0.143)	(0.139)
Reginoal variable	Uncontrol	Uncontrol	Uncontrol	Uncontrol	Control
R^2^	0.019	0.050	0.196	0.246	0.253
N	582	582	582	582	582

* p <  0.1, ^**^ p <  0.05, ^***^ p <  0.01

The results of control variables shows that specialization level, technical communications with feed companies, and the frequency of technical training significantly enhance production efficiency. Firstly, farmers with higher specialization accumulate more expertise. This leads to better management, improved breeding, and efficient resource use, ultimately boosting productivity [77]. Secondly, expertise from feed companies optimizes feed formulations, improving feed efficiency, reducing costs, and accelerating growth rates. This technical support also provides solutions to specific issues, enabling timely adjustments to enhance overall efficiency [78]. Lastly, social interactions and experiences significantly impact production efficiency. More interactions facilitate knowledge exchange and experience sharing, helping farmers learn from others’ successes and avoid common mistakes. Collaborations on bulk feed purchases and joint marketing enhance bargaining power and generate economies of scale [79].

### 5.2. Propensity score matching results

#### 5.2.1. Matching distribution of samples.

**Fig 3:**
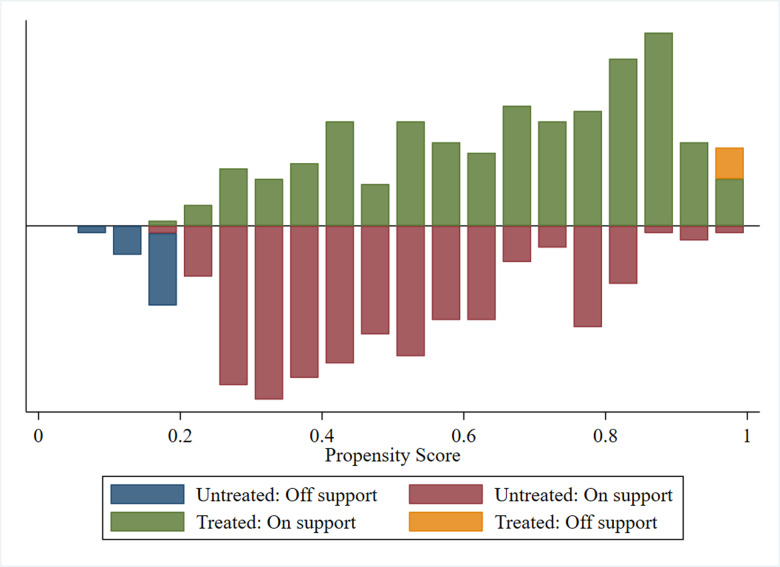
Propensity score distribution and common support for propensity score estimation.

This paper utilizes three distinct matching techniques: k-nearest neighbor matching, caliper matching, and kernel matching, to derive the sample’s maximum loss results, as shown in Fig 3, demonstrates that the sample loss is relatively minimal, indicating the effectiveness of the propensity score matching method used in this paper.

#### 5.2.2. Matching quality.

This paper evaluates the quality of the matching based on the distribution of explanatory variables before and after propensity score matching for farmers who participated in swine insurance and those who did not. However, this method alone cannot accurately estimate the probability of farmers participating in swine insurance. Therefore, a balance test was conducted on the matching variables, with the results presented in Table 4. The results show that, after propensity score matching, the standard deviations of most explanatory variables were significantly reduced and remained low. This indicates that there are no significant differences in the explanatory variables between the treatment and control groups after matching.

**Table 4:** Balance test results.

Matching method	Pseudo R^2^	LR Statistic	P-Value	Mean Bias (%)	Median Bias (%)
Before Matching	0.168	110.420	0.000	20.500	19.100
K-nearest Neighbor matching	0.083	63.210	0.000	12.500	12.400
Caliper Matching	0.037	28.170	0.030	9.200	7.400
Kernel Matching	0.039	29.610	0.020	9.100	9.200
Average	0.053	40.330	0.017	10.300	9.700

#### 5.2.3. Matching results.

Table 5 shows the impact of participating in swine insurance on farmers’ production efficiency. The results indicate that after incorporates regional variable, the matching outcomes are very similar across the three different matching methods used. Additionally, the ATT values are significant at the 1% statistical level, demonstrating the consistency of the estimates and the robustness of the paper’s findings.

**Table 5:** ATT effects on swine production efficiency for farmers participating in swine insurance.

Matching method	Treatment Group	Control Group	Difference	Standard Error
K-nearest Neighbor matching	0.392	0.344	0.048***	0.018
Caliper Matching	0.392	0.348	0.044***	0.014
Kernel Matching	0.392	0.344	0.048***	0.014
Average	0.392	0.345	0.047	0.015

*** p < 0.01

Overall, farmers’ participation in swine insurance can effectively improve swine production efficiency. The ATT effect is 0.047, which means compared to uninsured farmers, those who participate in swine insurance exhibit a 4.7% improvement in production efficiency. The empirical results clearly demonstrate the positive impact of swine insurance on production efficiency, Hypothesis 1 is validated. Theoretically, participation in swine insurance provides farmers with stable expected income and economic compensation in case of disasters, incentivizing them to engage in swine insurance. Additionally, swine insurance can enhance production efficiency by promoting technology adoption, expanding operational scale, and increasing capital investment.

### 5.3. Robustness tests

This paper conducts the following four robustness tests to verify the validity of the results: (1) replace the measure of key variables, (2) replace the matching method (3) data truncation (4) conducting an instrumental-variables test. The results affirm the robustness of the results.

#### 5.3.1. Replace the measurement of key variables.

This paper substitutes the measurement of the swine production efficiency, replace the output variable with the total weight of swine, which is obtained by subtracting the weight of piglets from the production of the main product [80]. According to Table 6, participation in swine insurance has a statistically significant positive impact on production efficiency at the 1% level. This finding suggests that swine insurance positively affects various dimensions of production efficiency, confirming the robustness of the results.

**Table 6:** Results of replacement of the measure of swine production efficiency.

Matching method	Treatment Group	Control Group	Difference	Robust standard error
K-nearest Neighbor Matching	0.373	0.336	0.037***	0.015
Caliper Matching	0.373	0.336	0.037***	0.012
Kernel Matching	0.374	0.334	0.039***	0.012

*** p <  0.01.

#### 5.3.2. Replace the matching method.

This paper replaces different matching methods to further validate the effect of the swine insurance participation influence the production efficiency. Table 7 displays the robustness test results obtained by using different matching methods. It demonstrates that after substituting Radius Matching, Near Neighbour Matching:k = 4 and Local Linear Matching, the ATT values remain significant at the 1% level. This consistency across different matching methods indicates robust estimation results and reliable findings.

**Table 7:** Results of replacement of different matching methods.

Matching method	Treatment Group	Control Group	Difference	Robust standard error
Radius Matching	0.392	0.345	0.048***	0.014
Near neighbour Matching	0.392	0.348	0.043***	0.015
Local Linear Matching	0.392	0.346	0. 045***	0.018

*** p <  0.01.

#### 5.3.3. Data truncation.

To reduce the impact of possible extreme values on the results of the regression analysis, this paper applies a 1% bilateral truncation to three variables susceptible to such extremes: age, farming years, and specialization level. The results are shown in Table 8. After making this adjustment, Table 8 indicates that swine insurance participation still positively impacts production efficiency, remaining statistically significant at the 1% level, thereby affirming the robustness of the baseline model’s findings.

**Table 8:** Results of data truncation.

	Treatment Group	Control Group	Difference	Robust standard error	N
Age	0.390***	0.331	0.059	0.016	474
Farming years	0.393***	0. 338	0.054	0.017	476
Specialization level	0.393***	0.343	0.049	0.016	477

*** p <  0.01.

#### 5.3.4. Endogeneity test.

To solve the possible endogenous problem between swine insurance participation and production efficiency, this paper adopts insurance awareness, which is how much farmers know about this risk-reducing policy as the instrumental variable for the endogeneity test. According to Xie (2024) [50], the awareness level of swine insurance, which is the degree of how much farmers know about the swine insurance, affects the participation of it, the more farmers know about the insurance, the stronger they are willing to participate. However, whether or not they know about the insurance does not affect the production efficiency, therefore, this paper adopts insurance awareness as an instrumental variable. In this paper, farmers’ awareness of insurance was categorized into five distinct levels: the lowest, lower, neutral, higher and the highest. These categories were presented in the questionnaire, and participants were instructed to select the option that most accurately reflected their understanding of swine insurance. Table 9 displays the results from the two-stage estimation with IV-2SLS. The DWH exogeneity test indicates that, at the 1% significance level, the assumption of all explanatory variables being exogenous is rejected, indicating endogeneity. With a first-stage F-value of 34.029, which exceeds 10, the absence of a weak instrumental variable issue confirms the validity of the instrument. The second-stage estimation results show that swine insurance participation positively impacts production efficiency, with statistical significance at the 5% level. This demonstrates the robustness of the findings, even after addressing endogeneity.

**Table 9:** Results of endogeneity test.

	(1) IV-2SLS Phase I	(2) IV-2SLS Phase II
Instrument variable	0.094***	
	(0.016)	
Insurance participation		0.124**
		(0.057)
Constant	0.064	0.080
	(0.246)	(0.132)
Control variables	Control	Control
Regional variable	Control	Control
F	34.029***	
Wald/DWH	186.34***	
N	582	

** p <  0.05, ^***^ p <  0.01.

### 5.4. Intermediary mechanism

As previously discussed, factors such as risk appetite, production investment, and technology adoption could have indirect effects on both swine insurance participation and production efficiency. To examine these effects, the study applies the approach detailed by Jiang (2022) [76], and the results are shown in Table 10. Regressions 1, 2, and 3 indicate the mediating effects of risk appetite, production investment and technology adoption, respectively. The results show that all three mediating variables have statistically significant effects at the 1% levels.

**Table 10:** Results of testing the mechanism of swine insurance participation in influencing the swine production efficiency.

	Regression (1)	Regression (2)	Regression (3)
	Risk appetite	Production investment	Technology adoption
Swine insurance participation	0.229***	0.256***	2.084***
	(0.039)	(0.079)	(0.190)
Constant	1.095***	8.803***	-0.122
	(0.317)	(0.464)	(1.073)
Control variables	Control	Control	Control
Regional control	Control	Control	Control
R^2^	0.692	0.516	0.347
N	582	582	582

*** p <  0.01.

Regression (1) reveals that swine insurance participation positively influences farmers’ risk appetite. The findings suggest that insured farmers are more likely to take risks in their decision-making, driven by the security that insurance provides against potential financial losses. This enhanced risk tolerance encourages farmers to pursue new opportunities and make more assertive production decisions. These results strongly support Hypothesis 2 (H2), which proposes that participation in swine insurance can improve production efficiency by boosting farmers’ risk appetite.

Regression (2) demonstrates that swine insurance significantly influences production investment. The results indicate that insured farmers are more proactive in their production decisions, such as increasing financial inputs and expanding production scale. This can be attributed to the reduced financial constraints and improved access to credit facilitated by insurance. With risks mitigated, farmers are more confident in investing additional resources, thereby enhancing overall production efficiency. This supports Hypothesis 3 (H3), which suggests that swine insurance participation can increase production efficiency by promoting greater investment in production inputs.

Regression (3) shows that participation in swine insurance significantly enhances the adoption of new technologies among farmers. Insured farmers are more likely to embrace advanced technologies, driven by the reduced perceived risks and financial security provided by insurance. This support lowers the barriers to investing in high-return but potentially risky innovations. By encouraging the adoption of modern technologies, swine insurance helps modernize and specialize swine production, ultimately boosting productivity and efficiency. These findings robustly support Hypothesis 4 (H4), which suggests that swine insurance participation can improve production efficiency by fostering the adoption of new technologies.

### 5.5. Heterogeneity analysis

Previous studies have emphasized the importance of farming scale in boosting swine production efficiency [8–10]. Large-scale operations enhance scale efficiency and reduce per-unit production costs. This shift promotes specialization and mechanization in farming, leading to more standardized production processes and higher resource utilization efficiency, thereby improving overall farming production efficiency. Expanding on these findings, the paper performs a regression analysis that incorporates an interaction term between farming scale and the core explanatory variables. The results, shown in Table 11, indicate that the coefficient of the interaction term is positive and significant at the 10% level, implying that the positive effect of swine insurance on production efficiency becomes more significant as the scale of swine production increases.

**Table 11:** Results of testing the mechanism of swine insurance participation in influencing the swine production efficiency.

	Swine production efficiency
Swine insurance participation	0.021*
	(0.011)
Scale of farming	0.010
	(0.006)
Swine insurance participation*Scale of farming	0.017**
	(0.008)
Constant	0.431***
	(0.159)
Control variables	Control
Regional control	Control
R^2^	0.629
N	582

* p <  0.1, ^**^ p <  0.05, ^***^ p <  0.01.

### 5.6. Further analysis: swine insurance coverage levels and production efficiency

Previous research on the primary effects of swine and agricultural insurance has predominantly focused on the transition from non-participation to participation in swine insurance and its impact on production efficiency [32,44,50]. However, given the current state of agricultural insurance development in China, the main challenge has shifted from insurance coverage to the adequacy of coverage. Currently, the level of coverage in China is only one-fifth that of the United States, one-third that of Canada, and half that of Japan [81]. Consequently, research on the effects of swine insurance should evolve from examining the extensive margin (participation) to the intensive margin (coverage level) to address emerging issues in practice.

Although consensus has been reached on enhancing insurance coverage levels, there remains considerable debate on how to define and quantify this concept. Goodwin and Mahul [26] argue that the coverage level of crop insurance is the insured amount, which is the maximum potential compensation available to the insured. The Research Team on Agricultural Insurance Coverage Levels in China suggests that the analysis of agricultural insurance coverage should be divided into macro and micro perspectives [82]. From a macro perspective, the coverage level reflects the extent to which swine insurance provides risk protection for swine industry, which is the ratio of total insured amounts to the total production value of the swine industry. At the micro level, coverage can be further divided into breadth and depth. Coverage breadth refers to the proportion of insured swine relative to the total number of swine raised, represents the unit coverage capacity of agricultural insurance. While coverage depth indicates the extent of risk protection provided per unit of swine production value, which is the ratio of the insured amount per unit to the production value per unit [83].

**Fig 4:**
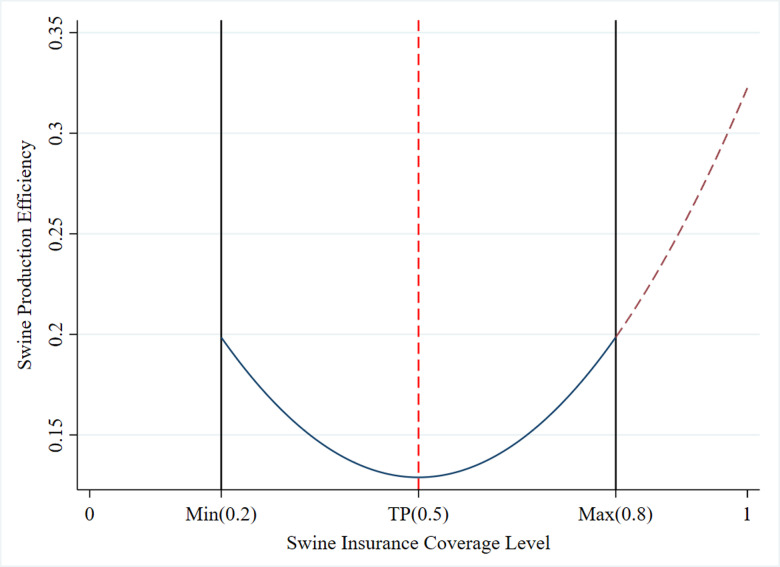
U-shaped relationship between swine insurance coverage level and production efficiency.

Given the research objectives of this paper, the coverage level should represent the risk protection capacity provided by swine insurance to farmers. Therefore, it is more appropriate to use the concept of coverage depth at the micro level. In this paper, the coverage level is defined as the ratio of the total insured amount to the total production value (total insured amount/total production value). This paper incorporates the level of swine insurance coverage into the analytical framework and analyzes its impact on production efficiency. The results are presented in Table 12 and Fig 4. Firstly, the analysis of the relationship between swine insurance coverage level and production efficiency reveals a significant U-shaped curve. roduction efficiency reveals a significant U-shaped curve. In Column (2), after introducing the squared term of the swine insurance coverage level, the coefficient of the linear term is significantly negative (-0.774), while the coefficient of the quadratic term is significantly positive (0.774), and are both significant, the calculated turning point is 0.5, falls within the valid range.This pattern of coefficients, characterized by a negative linear term and a positive quadratic term, confirms the U-shaped relationship between swine insurance coverage level and production efficiency. Secondly, the turning point occurs at an insurance coverage level of approximately 0.5, where production efficiency reaches its lowest point. Before this turning point, production efficiency decreases as the coverage level increases. After the turning point, production efficiency increases with higher coverage levels.

**Table 12:** Swine insurance coverage impact on production efficiency.

	(1) Swine production efficiency	(2) Swine production efficiency
Level	−0.176 *	−0.774***
	(0.102)	(0.254)
Square of Level		0.774**
		(0.302)
Constant	0.243	0.322
	(0.255)	(0.250)
Control variables	Control	Control
Regional variable	Control	Control
N	380.000	380.000
R^2^	0.250	0.253

*p <  0.1, ** p <  0.05, *** p <  0.01.

## 6. Discussion

This paper takes a micro-level perspective look at individual swine farmers to examine the impact of swine insurance on production efficiency and the underlying processes. Using representative household data from key swine-producing regions in Liaoning Province, China, the study employs statistical techniques, including propensity score matching and mediation models, to demonstrate that swine insurance enhances production efficiency. The positive impact of swine insurance is especially pronounced in larger-scale farms, which benefit from economies of scale, higher resource efficiency, and the adoption of advanced technologies. Additionally, this paper reveals a U-shaped relationship between insurance coverage and production efficiency.

Prior research has not specifically examined the relationship between swine insurance and production efficiency. Bulte and Lensink (2023) [84] found that agricultural insurance might reduce crop production efficiency in low-income countries due to imperfect input markets and the high cost of formal contracting, suggesting that informal institutions should receive more attention. These findings may be due to farmers’ lack of knowledge, limited access to credit, and low agricultural insurance uptake rates (below 10%). In contrast, our findings diverge from Bulte and Lensink’s conclusions. This discrepancy can be attributed to several factors unique to China. Firstly, China’s strong government provides substantial policy support and financial subsidies, resulting in a high uptake rate for agricultural insurance. Secondly, China’s large population and limited land resources necessitate the extensive use of technology to boost swine productivity. Additionally, long-term government advocacy and education have improved Chinese farmers’ educational levels, increasing their awareness of swine insurance’s importance and their willingness to participate in insurance programs.

Moreover, the heterogeneity analysis shows that the positive impact of swine insurance on production efficiency is more pronounced in larger-scale farms. This aligns with Cai et al., (2009) [32] previous studies, which indicating that formal insurance significantly increases farmers’ tendency to raise more sows. However, previous research has only revealed the positive impact of insurance participation on large-scale farming, without further investigating whether this effect influences swine production efficiency. This paper addresses this gap by conducting a more in-depth analysis. Specifically, firstly, large-scale farms can procure feed, medications, and other inputs in bulk at lower prices, thereby reducing production costs. Secondly, the utilization rate of machinery and infrastructure is higher in large-scale farms, which spreads fixed costs over a larger number of units, enhancing resource efficiency. Thirdly, large farms are more capable of investing in advanced technologies such as automated feeding systems and environmental control systems, which significantly improve production efficiency.

Additionally, this paper indicates a U-shaped relationship between swine insurance coverage level and production efficiency. The reasons include: firstly, at low coverage levels (0.2), farmers cannot fully rely on insurance to manage risks. Consequently, they adopt self-protection measures and more cautious production behaviors. They meticulously control costs and focus on disease prevention, selecting the most cost-effective technologies and equipment to maximize output. This precise investment and optimal resource allocation can enhance production efficiency. Secondly, at medium coverage levels (0.2-0.5), swine insurance provides basic risk protection. On one hand, due to moral hazard, farmers may develop a dependency on insurance, reducing their efforts and enthusiasm in production management, which leads to decreased production efficiency. On the other hand, swine insurance helps stabilize expected income and smooth out income fluctuations, farmers begin adjusting costs and changing management and production strategies, which may initially increase costs and reduce production efficiency in the short term. Thirdly, at high coverage levels (0.5-0.8), as insurance coverage increases further, swine insurance offers more comprehensive risk protection. The research reveals that when the coverage level of swine insurance exceeds 0.5, production efficiency improves significantly. However, approximately 96.9% of the sampled farmers have not surpassed 0.5 coverage level, only 0.17% greater than 0.8 coverage level, so it is essential to improve insurance coverage level at this stage. Effective risk management can boost farmers’ motivation and stability, with insurance as a safeguard, farmers are confident in making significant investments and adopting new technologies, which promotes investment in efficiency-enhancing technologies and equipment, thereby improving production efficiency.

Although the research is based on data from a specific region in Liaoning province, the findings of this study can be broadly applicable. Liaoning province represents a rapidly growing area for swine production, encompassing both traditional small and medium-sized farms and newly established large-scale and specialized farms, these characteristics make the data representative, the findings of this study can be applied to other regions, farmers of varying scales, and different agricultural environments. Previous research across 31 provinces in China has demonstrated that crop insurance improves production efficiency [85]. Therefore, the findings in the research are align with existing literature. Additionally, our sampling methodology is scientifically sound, and the study design various control variables and heterogeneity analyses to account for factors that may influence the results. The reproducibility of research findings is crucial for enhancing credibility and reliability. The conclusions can provide stronger evidence for agricultural policymakers if other research could replicate and obtain consistent results in different regions. This is especially relevant for improving and designing more effective swine insurance policies to enhance production efficiency, serve as valuable references for the development of agricultural insurance systems in other countries or regions. The heterogeneity analysis in the research confirms that the impact of swine insurance on production efficiency is present across farms of different scales. The survey of the research reveals a close relationship between the farming environment and the scale of operations. Large-scale farmers typically benefit from more advanced technology and superior management practices, whereas smaller-scale farmers often operate under less sophisticated conditions. Consequently, the scale-based heterogeneity analysis supports the generalizability of this study’s conclusions across diverse agricultural environments. This broader applicability is significant because it ensures that the findings can inform a wide range of policy decisions and agricultural practices, including the design and implementation of insurance schemes for dairy cattle, meat sheep, and seafood products, provide policymakers with robust evidence to guide the development of effective agricultural insurance policies.

Although the study presents important findings, it has limitations. (1) The results may be limited in regional relevance due to the small survey area. To improve generalizability, future research should include a broader geographic range covering more provinces and cities, allowing for a more balanced investigation of swine production efficiency under different factors, including varied swine insurance policies. Increasing the geographic variety of the sample would further strengthen the robustness of the findings. (2) This study did not investigate how different types of swine insurance, such as sow insurance, price insurance, or insurance combined with futures affect production efficiency. Future research should explore these variations in more detail to understand how each type of insurance uniquely influences production outcomes. (3) Swine production efficiency is a dynamic process. This study is limited by its use of cross-sectional data and does not address the dynamic evolution of swine production efficiency. Future research could focus on the temporal dynamics of the impact of swine insurance on production efficiency, providing insights into how these effects change over time.

## 7. Conclusions and policy implications

Swine insurance is essential for addressing the risks involved in swine production, significantly contributing to the advancement of the modern swine industry. This paper leverages the implementation of the swine insurance policy as a “quasi-natural experiment,” utilizing micro-survey data from 582 swine farmers in Liaoning Province, China, collected in 2023. The paper employs the SBM model to measure swine production efficiency and the PSM method to empirically examine the impact of swine insurance participation on production efficiency. The specific results of the study are outlined below:

Firstly, according to PSM method, compared to uninsured farmers, those who participate in swine insurance exhibit a 4.7% improvement in production efficiency, this conclusion is supported by robustness tests. The analysis of control variables shows that specialization level, technical communications with feed companies, and the frequency of technical training significantly also enhance production efficiency.

Secondly, the mechanism analysis demonstrates that swine insurance participation improves the production efficiency through risk appetite, production decision and technology adoption. Swine insurance enhances their risk appetite, encouraging them to invest and expand their operations. It also reduces capital constraints by lowering risks and facilitating access to loans, enabling more substantial investments. Furthermore, it boosts technology adoption by allowing farmers to invest in advanced technologies that improve production efficiency.

Thirdly, the analysis of economic heterogeneity shows that the positive effects of swine insurance on production efficiency increase with the scale of the farming operation. Large-scale operations enhance scale efficiency and reduce per-unit production costs, which promotes specialization and mechanization in farming, leading to improvement in production efficiency.

Lastly, the analysis of the relationship between swine insurance coverage level and production efficiency reveals a significant U-shaped curve. The turning point occurs at an insurance coverage level of approximately 0.5, where production efficiency reaches its lowest point. Before this turning point, production efficiency decreases as the coverage level increases. After the turning point, production efficiency increases with higher coverage levels.

From the perspective of swine insurance, research into swine production efficiency offers valuable insights for policymakers. It encourages the government to improve swine insurance policies and boosts the overall production efficiency of the swine industry, thereby carrying significant policy implications.

Firstly, enhancing policy support for swine insurance. To strengthen the resilience of swine farmers against market fluctuations and disease risks, the government should intensify policy support. Specific measures include expanding coverage and increasing compensation standards to alleviate the economic pressures faced by farmers, thereby improving their risk management capabilities and production efficiency. Given the diversity of farmers in terms of scale, income, and regional risks, insurance policies should be tailored to meet individual needs. Drawing on the experiences of countries with advanced livestock insurance systems, such as the United States and New Zealand, diverse products like index insurance and revenue insurance could be applied. If challenges arise in formulating these differentiated and diversified insurance policies, big data and artificial intelligence can be utilized to establish a comprehensive database. This approach allows for the integration of factors such as local climate, disease outbreaks, and market prices, enabling the development of more precise insurance solutions. A well-developed and efficient insurance system would not only promote the sustainable development of China’s swine industry but also enhance its competitiveness in international markets, providing robust support for meeting both domestic and global demand.

Secondly, enhancing the coverage level of swine insurance. The research reveals that when the coverage level of swine insurance exceeds 0.5, production efficiency improves significantly. However, approximately 97.6% of the sampled farmers have not surpassed this threshold. To address this, it is recommended to dynamically adjust insurance coverage amounts in line with annual changes in swine production costs, ensuring increased coverage to enhance the attractiveness of insurance products while avoiding moral hazards. Furthermore, a multi-layered risk dispersion system should be developed, which involve collaborating with “insurance + futures” model, and establishing a disaster risk fund to effectively diversify risks and strengthen the resilience of the insurance system. Given potential fiscal pressures on government subsidies, tax incentives could be used to encourage participation from commercial insurance companies, creating a cost-sharing mechanism among the government, insurers, and farmers. Additionally, establishing a swine insurance fund by pooling resources from multiple stakeholders could provide stable financial support for the insurance system, ensuring the stability of swine production.

Thirdly, enhancing credit support and promoting farmers’ adoption of technology. Rural financial institutions should increase credit support, particularly for smallholder farmers. Providing subsidized interest loans through fiscal support can help reduce borrowing costs, incentivize production expansion, and improve efficiency. Given the limited availability of collateral in rural areas, it is recommended to expand the scope of acceptable collateral, innovate credit models, and establish risk-sharing and information transmission mechanisms to develop effective credit screening technologies. At the same time, government and insurance companies should implement continuous education and training programs to enhance farmers’ expertise in modern swine farming techniques, disease prevention, and management skills. Advanced technologies such as automated feeding systems and environmental control technologies should be promoted to encourage specialization and modernization in swine farming, thereby improving production efficiency.

Lastly, promoting appropriately scaled farming operations. While large-scale swine farming is on the rise, small-scale farming remains prevalent in China. In 2021, there were 137,000 large-scale farms in China, accounting for only 0.06% of the total number of farmers. To address this, targeted measures such as subsidies, training, and market support are recommended to help small and medium-sized farmers improve infrastructure and expand their operations. Additionally, fostering partnerships between new agricultural entities and small-scale farmers can strengthen specialized production capabilities and market competitiveness. However, the dual challenges of growth and transformation are intensifying resource supply-demand imbalances and environmental pollution issues. Thus, it is imperative to establish an ecological compensation mechanism and develop a multi-tiered green finance system, incorporating green credit, green bonds, and green insurance, to support environmentally sustainable farming practices.

## Supporting information

S1 File Supporting survey data. (XLSX)
